# Structural changes induced by heating affect ovalbumin-antigen processing and reduce allergic response in mouse model of food allergy

**DOI:** 10.1186/2045-7022-4-S2-P25

**Published:** 2014-03-17

**Authors:** Jaroslav Goliáš, Martin Schwarzer, Hana Kozáková, Dagmar Šrùtková, Ludmila Tuèková

**Affiliations:** 1 Institute of Microbiology, The Academy of Sciences of the Czech Republic, Department of Immunology and Gnotobiology, Prague, Czech Republic; 2 Institute of Microbiology, The Academy of Sciences of the Czech Republic, Department of Immunology and Gnotobiology, Nový Hrádek, Czech Republic

## Background

Food allergy is a serious health concern affecting about 8% of young children and about 3% of adults. The egg protein ovalbumin belongs among six frequent food allergens. Our goal was to develop the mouse model of ovalbumin food allergy and investigate how thermal processing affects its ability to induce allergic responses.

## Methods

Effect of thermal processing (70°C and 95°C) on an ovalbumin secondary structure was characterized by circular dichroism and by kinetics of pepsin digestion with subsequent HPLC analysis. BALB/c mice were sensitized intraperitoneally and challenged intragastrically with native form or thermal processed ovalbumin (70°C). Clinical symptoms (allergic diarrhea, weight and temperature) were observed during the mice challenging. Levels of allergen-specific serum antibodies were determined by ELISA (IgA, IgG1 and IgG2a) by beta-hexosaminidase release test (IgE). Cytokine production (IL-4, IL-5, IL-6, IL-10, IL-13, IL-17, IFN-gamma) and mast cell protease-1 activity were assessed by ELISA. The activities of brush-border hydrolases were determined in the jejunum of treated mice.

## Results

Heating of ovalbumin caused slight irreversible changes in secondary structure and significantly decreased clinical symptoms (allergic diarrhea). The thermal processing also decreased immune allergic response on the level of IgE, IL-4, IL-5, IL-13. Furthermore, thermal-processed ovalbumin induced lower activities of serum mast cell protease-1 and enterocyte brush border membrane alkaline phosphatase as compared to native ovalbumin. On the other hand, thermal-processed ovalbumin stimulated higher IgG2a antibodies in sera and IFN-gamma secretion by splenocytes.

## Conclusion

In our mice model of food allergy, we show that even a slight change in the secondary structure of ovalbumin after thermal processing reduces its allergenicity and induces shift towards Th1 immune responses. At present, we utilize this model for studying of food allergy in germ-free mice.

